# First-Principles Study on Mechanical, Electronic, and Magnetic Properties of Room Temperature Ferromagnetic Half-Metal MnNCl Monolayer

**DOI:** 10.3390/nano13111712

**Published:** 2023-05-23

**Authors:** Yuxin Zou, Xin Wang, Liwei Liu, Tielei Song, Zhifeng Liu, Xin Cui

**Affiliations:** School of Physical Science and Technology, Inner Mongolia University, Hohhot 010021, China

**Keywords:** two-dimensional (2D) ferromagnetic materials, half-metal, mechanical properties, biaxial strain, first principles

## Abstract

Two-dimensional ferromagnetic (FM) half-metals are highly desirable for the development of multifunctional spintronic nano-devices due to their 100% spin polarization and possible interesting single-spin electronic states. Herein, using first-principles calculations based on density functional theory (DFT) with the Perdew–Burke–Ernzerhof (PBE) functional, we demonstrate that the MnNCl monolayer is a promising FM half-metal for spintronics. Specifically, we systematically investigated its mechanical, magnetic, and electronic properties. The results reveal that the MnNCl monolayer has superb mechanic, dynamic, and thermal (ab initio molecular dynamics (AIMD) simulation at 900 K) stability. More importantly, its intrinsic FM ground state has a large magnetic moment (6.16 μ_B_), a large magnet anisotropy energy (184.5 μeV), an ultra-high Curie temperature (952 K), and a wide direct band gap (3.10 eV) in the spin-down channel. Furthermore, by applying biaxial strain, the MnNCl monolayer can still maintain its half-metallic properties and shows an enhancement of magnetic properties. These findings establish a promising new two-dimensional (2D) magnetic half-metal material, which should expand the library of 2D magnetic materials.

## 1. Introduction

Owing to their ferromagnetic (FM) order and unique electronic properties with 100% spin polarization [1], two-dimensional (2D) FM half-metal materials have attracted extensive attention in the past few decades [2,3], providing new opportunities for the development of multifunctional spintronic devices [4,5]. In practical applications, the 2D FM half-metals with large magnetocrystalline anisotropic energies and high Curie temperatures are always highly desirable.

According to the Mermin Wagner theorem [6], magnetic crystalline anisotropy (MCA) is the key to generating a long-range magnetic order in 2D materials by breaking the Hamiltonian continuous spin symmetry. In addition, the MCA contributes to the stabilization of magnetic data storage, keeping the magnetic moment direction free from thermal fluctuations [7]. Obviously, the MCA is a key factor for 2D intrinsic ferromagnetism, which can be characterized by magnetic anisotropic energy (MAE). A larger MAE is better for resisting thermal fluctuations.

In 2017, the experimental discovery of 2D intrinsic ferromagnets, namely monolayer CrI_3_ and bilayer Cr_2_Ge_2_Te_6_, triggered a research boom of 2D FM materials [8,9]. However, their applications in high-density magnetic storage and spintronic devices are greatly limited by their low Curie temperature (*T*_C_) [10].

At present, the search for new FM half-metal materials with high MAE and *T*_C_ has become a hot research direction for the development of 2D spintronics [11,12,13,14,15,16,17,18,19,20,21,22]. On the other hand, some methods have been designed to improve the magnetic properties of the discovered 2D FM materials, which includes applying an external field [16], carrier doping [23,24], and strain engineering [25].

As an emerging category of 2D FM half-metal materials [26], 2D quasi-planar transition metal nitrides with small fluctuations have received much attention in recent years [7,26,27,28]. For instance, it has been proposed that the MnP and MnAs monolayers hold an intrinsic FM half-metallic ground state and have remarkably large MAE (166 μeV and 281 μeV for MnP and MnAs, respectively) and high *T*_C_ (495 and 711 K) [7]. Moreover, the related Janus structures (e.g., Mn_2_PAs) have also been proven to be FM half-metallic, and their *T_C_* and MAE are calculated to be 557 K and 148.5 μeV, respectively [27]. In addition, this family can be extended to include halogen elements, such as the half-metallic MnNF monolayer and the MnNBr monolayer with intrinsic ferromagnetism, also exhibiting novel topological quantum states near the Fermi surface [21,28]. Strain engineering is an effective approach to tune the physical properties of nanomaterials. It has been demonstrated that external strain can tune the electronic energy band gap, affect magnetic anisotropy, and cause phase transition [29].

Inspired by these, using first-principles calculations, we take the isoelectronic system, the MnNCl monolayer, as the object of our study, so as to systematically investigate its mechanical, magnetic, and electronic properties; then, we further examine the strain effect on these properties. The results show that the MnNCl monolayer has dynamic and thermal stability (up to 900 K). Moreover, it has good mechanical properties, large magnetic moments (6.16 μ_B_), large MAE (184.5 μeV), an ultra-high *T*_C_ (952 K), and a wide direct band gap (3.10 eV) in the spin-down channel. By applying biaxial strains, the MnNCl monolayer can effectively increase its magnetic properties and modulate its electronic properties.

## 2. Computational Methods

In this work, first-principles calculations based on density functional theory (DFT) [30,31] were performed in the Vienna Ab initio Simulation Package (VASP) [32,33] with the Perdew–Burke–Ernzerhof (PBE) functional in the generalized gradient approximation (GGA) [34]. The ion–electron interactions were described by the projector-augmented wave (PAW) method [35,36]. The cut-off energy of the plane wave was set to 500 eV. To avoid adjacent interaction, a vacuum region of 25 Å was added along the [001] directions. The convergence criteria of total energy and force were set to 10^−8^ eV and 0.001 eV/Å, respectively. The Γ-centered Monkhorst–Pack method with a uniform density of 2π × 0.01 Å^−1^ was used to sample the 2D Brillouin zone (BZ) [37]. Due to the strong correlation interaction of 3*d* electrons in the Mn atom, the GGA + U correction was used in the calculation of the electronic and magnetic properties [38,39]. The Coulomb interaction parameter U of the Mn 3*d* electrons was assumed to be 4.0 eV, which was consistent with the previously reported values [7,27]. The spin-orbit coupling (SOC) interactions, known to be important to determine the orbital magnetic moment and MAE, were considered in relativistic non-collinear calculations (DFT + U + SOC) with different quantization axes. In order to examine the dynamic stability of our proposed system, phonon spectra were calculated using density functional perturbation theory embedded in the PHONOPY codes [40]. A 4 × 4 × 1 supercell was used to perform the ab initio molecular dynamics (AIMD) simulation at 900 K so as to confirm the thermal stability of the system.

## 3. Results

### 3.1. Atomic Structure

**Figure 1:**
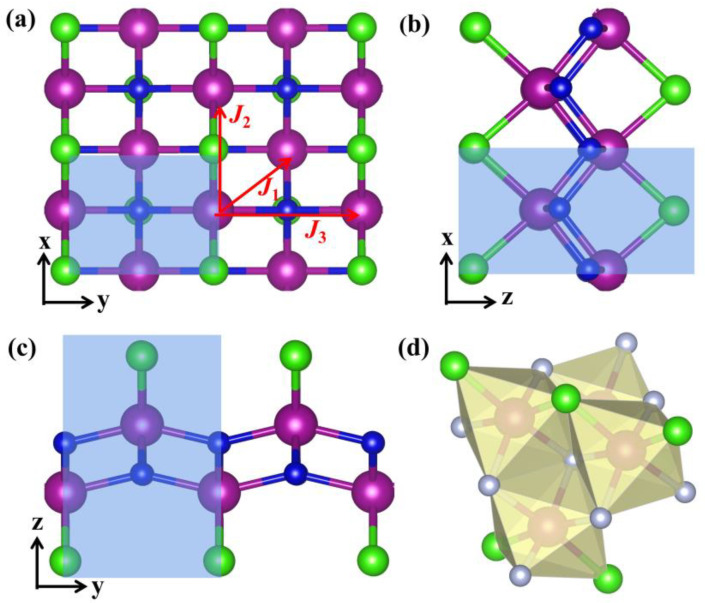
Atomic structure of the MnNCl monolayer from different directions: view from lattice vector (**a**) *z*, (**b**) *y*, and (**c**) *x*. The purple, blue, and green balls denote Mn, N, and Cl atoms, respectively. The area marked by blue is the corresponding primitive cell. In (**a**), the labeled *J*_1_, *J*_2_, and *J*_3_ represent the first, second, and third neighbor exchange paraments between Mn–Mn atoms, respectively. (**d**) Octahedron of four N and two Cl atoms surrounding the Mn atom.

In Figure 1, different views of the 2 × 2 × 1 supercell structure of the 2D MnNCl monolayer are presented. In the side view, one can see that the MnNCl monolayer is a quasi-planar structure with small fluctuations in the *z*-axis. The optimized MnNCl monolayer possesses an orthorhombic Bravais lattice with Pmmn symmetry. The equilibrium lattice constants are calculated to be 3.21 Å and 3.84 Å for *a* and *b*, respectively. Each primitive unit cell (see the shaded part in Figure 1) contains six atoms: two Mn atoms, two N atoms, and two Cl atoms. Every Mn atom is surrounded by a slightly distorted tetrahedron composed of four N atoms and two Cl atoms (Figure 1d). The Mn–N bond lengths are calculated to be 1.96 Å and 1.97 Å along the *x*- and *y*-axes of the lattice, respectively. Moreover, each Mn atom is adjacent to two Cl atoms, forming two Mn–Cl bonds with a distance of 2.34 Å.

### 3.2. Stabilities

**Figure 2:**
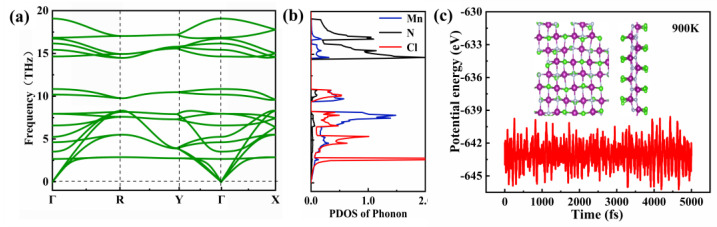
(**a**) Phonon dispersion spectra and (**b**) partial density of phonon states of the MnNCl monolayer. (**c**) The fluctuation of total potential energy of a 4 × 4 × 1 supercell for the MnNCl monolayer during first-principles molecular dynamic simulation at 900 K. The snapshot is the top and side views for the supercell at the end of the simulation.

To examine the dynamic stability of the MnNCl monolayer, the phonon spectra of the MnNCl monolayer are calculated. As shown in Figure 2a, there is no imaginary frequency mode in the whole Brillouin zone, indicating that the MnNCl monolayer is dynamically stable. From the partial phonon density of states, one can see that the low-frequency acoustic modes are mainly contributed by the heavier Mn and Cl atoms, while the high-frequency optical branches are mainly dominated by the lighter N atoms. In addition, to explore its mechanical stability, we further calculate the independent elastic constants of the MnNCl monolayer. The results are *C*_11_ = 113.273 Nm^−1^, *C*_12_ = 14.519 Nm^−1^, *C*_22_ = 141.676 Nm^−1^ and *C*_66_ = 48.502 Nm^−1^, which can well satisfy the Born–Huang criterion, i.e., *C*_11_ > 0, *C*_11_*C*_22_ > $C_{12}^{2}$, and *C*_66_ > 0 [41]. Thus, the MnNCl monolayer should be mechanically stable.

To assess its thermal stability, which is crucial for its experimental fabrication and potential practical applications, we carry out ab initio molecular dynamic simulations [42,43] by building a 4 × 4 × 1 supercell of the MnNCl monolayer at 900 K. Using the canonical ensemble, the temperature in our simulation is controlled by the Nosé–Hoover thermostat [44]. As illustrated in Figure 2, the calculated total potential energies fluctuate in the vicinity of a fixed value (about −643.5 eV) with a very narrow energy window. This suggests that the MnNCl monolayer is also thermally stable above room temperature, at least up to 900 K.

### 3.3. Mechanical Properties

On the basis of the determination of its mechanical stability, we further perform an in-depth investigation for the mechanical properties of the MnNCl monolayer. As is known, Young’s modulus (*Y*) can reflect the flexibility or stiffness of materials. The maximum value of Young’s modulus for the MnNCl monolayer is evaluated to be 139.82 Nm^−1^, which is higher than that of the CrI_3_ monolayer (28.606 Nm^−1^), but lower than that of the graphene monolayer (342 Nm^−1^) [45,46]. Poisson’s ratio (*ν*) is the other important mechanical parameter, which can reflect the brittleness and ductility of a 2D material. The critical value is 0.33, which is usually used to distinguish brittleness from ductility [47]. For the MnNCl monolayer, the Poisson’s ratio is calculated to be 0.184, indicating that it is a brittle 2D material [48]. Furthermore, the shear modulus (*G*) can be applied to assess the resistance against the deformation caused by the shear stress for a 2D material. Our calculated result shows that the maximum shear modulus of the MnNCl monolayer is 55.77 Nm^−1^, which is higher than that of the CrI_3_ monolayer (11.61 Nm^−1^) [45]. Pugh’s modulus (*K* = *B*/*G*, *B* is the bulk modulus) can also be used to determine bonding nature; a low (high) *B*/*G* value is associated with brittleness (ductility). The Pugh’s ratio is calculated to be 1.35, which is lower than the critical value of approximately 1.75, indicating that the MnNCl monolayer can be classified as brittle material.

**Figure 3:**
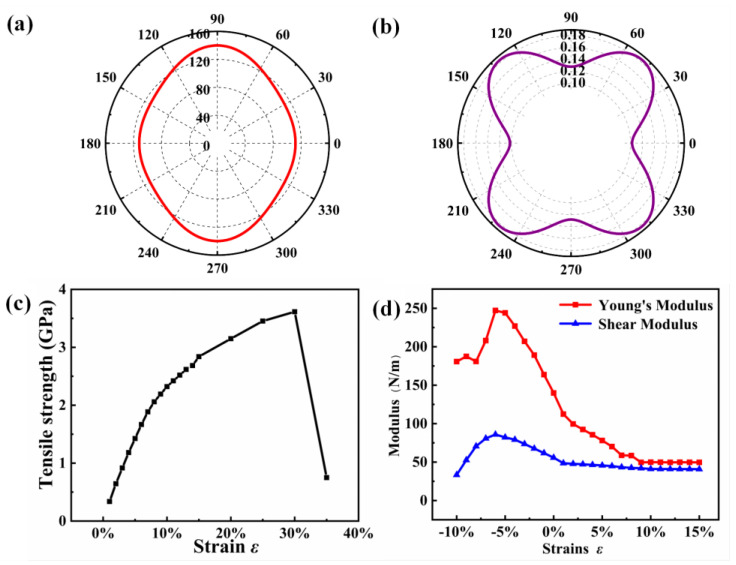
The in-plane (**a**) Young’s modulus, (**b**) Poisson’s ratio as functions of the arbitrary direction *θ* in the polar coordinates. (**c**) The dependence of the tensile strength with the strain of MnNCl monolayer. (**d**) Young’s modulus (red dotted line) and the shear modulus (blue dotted line) under biaxial strain.

The anisotropic mechanical properties of the MnNCl monolayer can be described comprehensively by calculating the distribution of mechanical moduli in the 2D polar coordinate system. The in-plane Young’s modulus and Poisson’s ratio as functions of the arbitrary direction *θ* (*θ* is the angle relative to the positive *x* direction) can be described as:

(1) $$Y=\frac{\Delta }{C_{11}s^{4}+C_{22}c^{4}+(\frac{\Delta }{C_{66}}-2C_{12})c^{2}s^{2}}$$

(2) $$\nu =-\frac{\left(C_{11}+C_{22}-\frac{\Delta }{C_{66}})c^{2}s^{2}-C_{12}(c^{4}+s^{4}\right)}{C_{11}s^{4}+C_{22}c^{4}+(\frac{\Delta }{C_{66}}-2C_{12})c^{2}s^{2}}$$

where $\Delta =C_{11}C_{22}-C_{12}^{2}$, c=cosθ, s=sinθ. The spatial distributions of Young’s modulus and Poisson’s ratio with respect to *θ* are displayed in Figure 3a and Figure 3b, respectively. On the whole, one can see that the Young’s modulus and the Poisson’s ratio of the MnNCl monolayer are highly anisotropic in the whole plane. Specifically, the Young’s modulus in the [010] direction is significantly larger than that in the [100] direction (Figure 3a), implying its strong resistivity against deformation caused by external forces in the [010] direction. From the distribution of Poisson’s ratio (see Figure 3b), one can find that the most brittle direction is [100] direction, while the most ductile direction is [110] direction.

To evaluate the ideal strength of the MnNCl monolayer, we calculate the tensile strength as a function of the biaxial strain. As is illustrated in Figure 3c, one can find when the strain is larger than 30% that the tensile stress of the MnNCl monolayer has a rapid descent, indicating that there is a breaking of the chemical bonds. Therefore, one can conclude that the ideal strength (*σ*), which is the first derivative of the total energy for the volume, of the MnNCl monolayer is 3.6 GPa, corresponding to 30% mechanical tensile strain. Moreover, we have also investigated the strain effect on the Young’s moduli and shear moduli. Figure 3d displays the calculated Young’s modulus and shear modulus for the biaxial strains ranging from −10% to 15%. They both first increase to a maximum value (247.17 Nm^−1^ for Young’s modulus and 85.86 Nm^−1^ for shear modulus) at −6% strain and then decrease. In other words, the material has a maximum Young’s modulus and shear modulus when compressed by 6%.

### 3.4. Magnetic Properties

**Figure 4:**
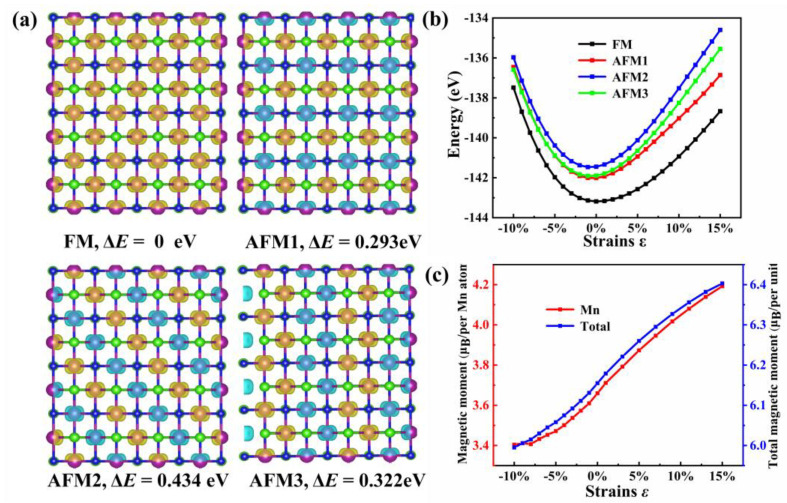
(**a**) The spatial distribution of spin-polarized electron density for the MnNCl monolayer in FM and possible AFM configurations. Δ*E* is the relative total energies with respect to FM configuration. Yellow and blue isosurfaces represent spin-up and spin-down densities, respectively. (**b**) The relative total energies of different magnetic states of the MnNCl monolayer as a function of biaxial strains from −10% to 15%. (**c**) Magnetic moment per unit(blue dotted line) and per Mn atom (red dotted line) as a function of the strain.

To determine the magnetic ground state of the MnNCl monolayer, we construct both FM and possible antiferromagnetic (AFM) magnetic structures for the supercells of 2 × 2 × 1, as shown in Figure 4a. Then, we perform spin-polarized DFT calculations. The calculated relative energy (Δ*E* = *E*_AFM_ − *E*_FM_) indicates that the FM state is much more stable than all the AFM states, having the lowest total energy (−143.185 eV). Therefore, the magnetic ground state of the MnNCl monolayer should be the FM state. Our analysis of the spatial distribution of the spin-polarized electron density further reveals that its large FM localized magnetic moment (6.16 μ_B_ per unit cell) is mainly contributed by Mn atoms.

To check whether the magnetic ground state of the MnNCl monolayer will be changed under external strains, we also calculate the total energies of different magnetic states as a function of biaxial strains ranging from −10% to 15% (see Figure 4b). The results demonstrate that the FM state of the MnNCl monolayer under strain is quite robust, which means that a mild external force cannot damage the orderly storage of magnetic data.

In addition, with the increase in the biaxis strain from −10% to 15%, the total magnetic moment per unit gradually increases from 5.999 μ_B_ to 6.403 μ_B_, and the main contribution still comes from the Mn atoms (see Figure 4c). This means that the magnetic moment can be enhanced under tensile biaxial strain.

MCA contributes to the thermal stability of magnetic data storage by insulating the magnetic moment direction from thermal fluctuations. In general, the MAE as a reflection of the MCA depends mainly on two elements: the spin-orbit coupling and the magnetostatic dipole–dipole interactions. However, the contribution of the magnetic dipole–dipole interaction is small and normally negligible [49]. Thus, it is defined as the energy required to shift the system’s magnetic moment from any other direction to the easy axis direction (the direction of lowest energy), which can be expressed as: *E*_MAE_ = *E*_M_other_ − *E*_M_easy_. The high MAE helps the FM order resist thermal fluctuations. GGA + U + SOC calculations are performed on the MnNCl monolayer to obtain the total energies along the [100], [010], [110], and [001] magnetic moment directions. The total energies in these directions are calculated to be −35.828495, −35.828620, −35.828577, and −35.828864 eV per unit cell, respectively. Thus, the easy axis direction is the [001] direction and the MAE of the [100], [010], and [110] directions are 184.5, 122.0, and 143.5 μeV per Mn atom, respectively. The maximum value of MAE (184.5 μeV) is significantly larger than the values of 166 μeV, 148.5 μeV, 130 μeV, 110 μeV, and 169 μeV in the MnP monolayer [7], Mn_2_PAs monolayer [27], Cr_2_PAs monolayer [50], CrOCl monolayers [51], and MnNF monolayer [28], respectively. To the best of our knowledge, such large MAE is rarely observed in 2D magnetic materials, which is greatly helpful for their practical applications above room temperature. Moreover, low-strain MAE can reflect the magnitude of magnetostriction constants [52,53,54,55,56,57,58], which are all produced by spin-orbit coupling.

The magnetostrictive coefficient *λ* can be obtained from the strain dependences of MAE as the following equations [52]:

(3) $$\lambda =-B_{1}/C_{11}$$

(4) $$B_{1}=\frac{3}{4}\frac{d\mathrm{MAE}}{d\varepsilon }$$

**Figure 5:**
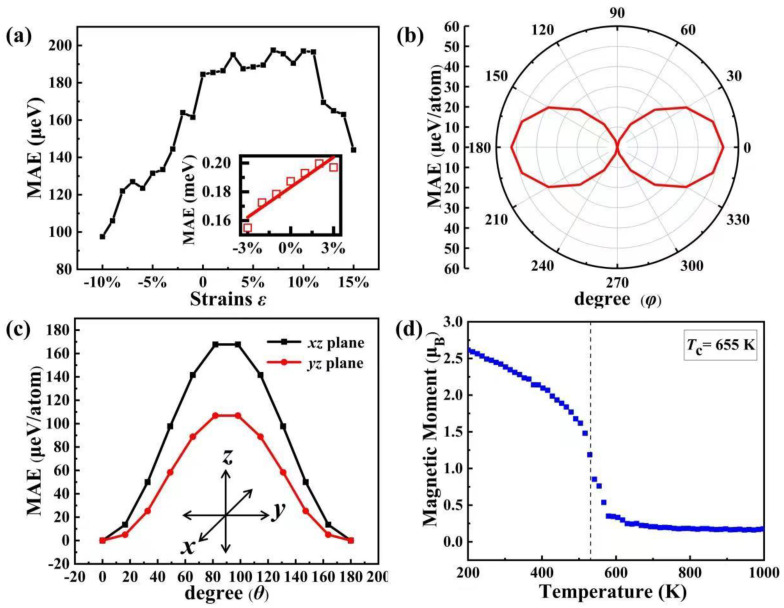
(**a**) MAE along the [100] direction as a function of biaxial strain; the snapshot is the linear fitting results of low-strain from −3% to 3%. (**b**) MAE projected in the (110) plane. (**c**) MAE projected in the (101) and (011) planes, represented by black and red lines, respectively. (**d**) Average magnetic moment of MnNCl as a function of temperature, the dashed line corresponds to Curie temperature *T*_C_.

Based on Equation (3) and the obtained low-strain MAE (see the snapshot in Figure 5a), the *λ* are calculated to be −5.2 ppm, which is much lower than that of two-dimensional Fe_3_GeTe_2_ [52].

The effect of biaxial strains ranging from −10% to 15% on MAE has also been explored. As shown in Figure 5a, MAE reaches its maximum value of 197.5 μeV under 7% tensile strain, which means that the tensile strain has an enhanced effect on the MAE, like the case of magnetic moment. To investigate the characteristics of MAE in whole space, we calculate the projections of MAE in the (110), (101) and (011) planes. As presented in Figure 5b, MAE shows significant anisotropy and has a hammer-like distribution in the (110) plane. The projection of MAE in the (101) plane and the (011) plane also shows significant anisotropy, and the MAE in the (101) plane is significantly larger than that in the (011) plane, as shown in Figure 5c. This suggests that the distribution of the MAE in the whole space can be described by a somewhat flattened spindle shape and presents anisotropy in the whole space.

The *T*_C_ of the MnNCl monolayer is estimated using the mean-field approximate (MFA) [59]. As presented in Figure 1a, each Mn atom has first, second, and third neighbor magnetic exchange interactions. Therefore, the Hamiltonian of the MnNCl monolayer in a Heisenberg model can be described as

(5) $$H=-\sum_{i,j}J_{1}M_{i}M_{j}-\sum_{k,l}J_{2}M_{k}M_{l}-\sum_{m,n}J_{3}M_{m}M_{n}$$

Here, *J*_1_, *J*_2_, and *J*_3_ correspond to the first, second, and third neighbor exchange parameters (see Figure 1a), respectively. *Mx* (*x* = *i*, *j*, *k*, *l*, *m*, *n*) present the spin magnetic moment on different sites. Therefore, the total energies of different magnetic configurations (as shown in Figure 4a) can be described by

(6) $$E_{\mathrm{FM}}=E_{0}-(4J_{1}+2J_{2}+2J_{3})M^{2}$$

(7) $$E_{\mathrm{AFM}1}=E_{0}-(-4J_{1}+2J_{2}+2J_{3})M^{2}$$

(8) $$E_{\mathrm{AFM}2}=E_{0}-(-2J_{2}-2J_{3})M^{2}$$

and

(9) $$E_{\mathrm{AFM}3}=E_{0}-(-2J_{2}+2J_{3})M^{2}$$

where *E*_0_ is the energy of the ground state. On the basis of these energies, the exchange parameters of *J*_1_, *J*_2_, and *J*_3_ can be calculated via

(10) $$J_{1}=-\frac{E_{\mathrm{FM}}-E_{\mathrm{AFM}1}}{8M^{2}}$$

(11) $$J_{2}=-\frac{E_{\mathrm{FM}}+E_{\mathrm{AFM}1}-2E_{\mathrm{AFM}3}}{8M^{2}}$$

and

(12) $$J_{3}=-\frac{E_{\mathrm{AFM}3}-E_{\mathrm{AFM}2}}{4M^{2}}$$

The calculated *J*_1_, *J*_2_ and *J*_3_ are 4.068, 4.885 and 3.100 meV per Mn atom, respectively. All exchange parameters are positive, implying that the first, second, and third neighbor interactions belong to the FM order.

According to the theory of statistical ensemble [11,21], the partition function of the magnetic moment *M* is solved as follows:

(13) $$Z=\sum_{m=-M,-M+2,\ldots ,M-2,M}\exp\left[\frac{(\gamma_{1}J_{1}+\gamma_{2}J_{2}+\gamma_{3}J_{3})m\langle M\rangle }{k_{B}T}\right]$$

where *γ*_1_, *γ*_2_, and *γ*_3_ are the first, second, and third neighbor coordination numbers of the magnetic atoms, respectively. For the proposed MnNCl monolayer, they are equal to 4, 2, and 2 for each Mn atom, respectively. Then, the statistical average of the magnetic moments can be solved from the following equation:

(14) $$\langle M\rangle =\frac{1}{Z}\sum_{m=-M,-M+2,\ldots ,M-2,M}m\times \exp\left[\frac{(\gamma_{1}J_{1}+\gamma_{2}J_{2}+\gamma_{3}J_{3})m\langle M\rangle }{k_{B}T}\right]$$

Here, we define

(15) $$P=\frac{\gamma_{1}J_{1}+\gamma_{2}J_{2}+\gamma_{3}J_{3}}{k_{B}T}$$

For *M* = 3 μ_B_, the <*M*> can thus be rewritten as

(16) $$\langle M\rangle =\frac{\sinh(P\langle M\rangle )+3\sinh(3P\langle M\rangle )}{\cosh(P\langle M\rangle )+\cosh(3P\langle M\rangle )}$$

In this case, the root of Equation (16) is *P* = 0.2. The <*M*> moving close to 0 implies that the magnetic moment changes from ferromagnetic to nonmagnetic order, and this critical temperature is the $T_{C}^{MFA}$

(17) $$T_{C}^{\mathrm{MFA}}=\frac{\gamma_{1}J_{1}+\gamma_{2}J_{2}+\gamma_{3}J_{3}}{Pk_{B}}$$

Because MFA generally overestimates *T*_C_, it can be modified by an empirical relation $T_{C}/T_{C}^{MFA}=0.51$ [11]. Therefore, the value of the *T*_C_ should be 952 K at equilibrium structure, and this value is larger than in some reported systems, such as the MnP (495 K), MnAs (711 K) [5], Mn_2_PAs (557 K) [25], CrSI (385 K) [60], MnNF (890 K) [26], and MnNBr (910 K) monolayers [19]. To verify this result, we further perform Monte Carlo simulations to obtain the Curie temperature. Figure 5d shows the results of average magnetic moments as a function of temperature, with the Curie temperature *T*_C_ estimated to be around 655 K. This high *T*_C_ indicates that the FM order of the MnNCl monolayer can be maintained over room temperature, which is quite helpful for its practical application in future spintronic devices.

### 3.5. Electronic Properties

**Figure 6:**
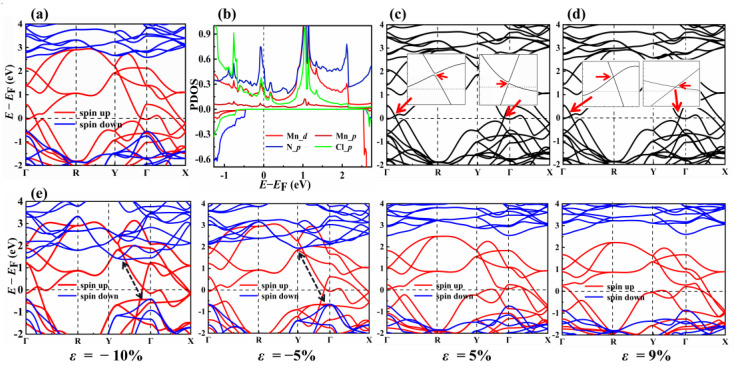
(**a**) Calculated 2D band structure of single-layer MnNCl at equilibrium state. (**b**) Spin-resolved projected density of states (PDOS). The band structures around Γ point calculated by the DFT + U + SOC method with (**c**) in-plane magnetization directions and (**d**) out-of-plane magnetization directions. (**e**) Calculated 2D band structure by DFT + U method under biaxial strain at ε = −10%, −5%, 5%, and 9%, respectively. The Fermi energy is set to zero; the band of spin-up and spin-down are highlighted by red and blue, respectively; the red arrows in subfigure (**c**,**d**) point out the positions of linear crossing; the black double-headed arrows in subfigure (**e**) point out the positions of CBM and VBM.

The band structure and density of states of the MnNCl monolayer are calculated to investigate its electronic properties. The Coulomb interaction parameter U, for Mn 3*d* electrons, is assumed to be 4 eV, which has been proven to be reliable for Mn atoms in previous works [5]. Figure 6a shows the spin-polarized band structure of the MnNCl monolayer with the GGA + U method. In the spin-up channel, the bands are metallic with half-filled bands crossing the Fermi energy. For the spin-down channel, the band exhibits semiconducting properties with a direct band gap of 3.10 eV, which is wide enough to prevent spin leakage [12]. Interestingly, there exist linear crossing bands near the Fermi level. Two Weyl states can be found along high symmetry paths from Γ to R and from Y to Γ (Figure 6a). These suggest that the proposed MnNCl monolayer is a 2D intrinsic FM half-metal with 100% electron-spin-polarization and massless Weyl fermions as conducting carriers, which is much needed for future ultra-high speed spintronic devices. To understand the electron properties near the Fermi level, the projected density of states (PDOS) of the MnNCl monolayer is calculated, as is shown in Figure 6b. The results show that the fully polarized metallic state near the Fermi level is mainly contributed by the Mn-*d*_x_^2^_−y_^2^, *d*_yz_, N-*p*_y_ and Cl-*p*_z_ orbitals.

To further explore whether the interesting linear crossing will open a band gap at the Weyl point under considering SOC, the band structure of the MnNCl monolayer is recalculated by using GGA + U + SOC with the spin orientations set to different magnetization orientations. For in-plane magnetization, our calculation shows that the SOC effect can induce a sizable band gap (~10 meV see Figure 6c) in both of the two band crossing points near the Fermi level. However, when the magnetization direction is tuned to out-of-plane, the two band crossing points near the Fermi surface have not been broken, as shown in Figure 6d. This means that the two band crossings can be well preserved under the out-of-plane SOC.

As is known, applying mechanical strains is a promising approach for engineering the properties of 2D materials. For this, we further investigate the modulation of the band structure under modest biaxial strains, ranging from −10% to 15%. From Figure 6e, it can be seen that the band structures have significant change due to the effects of strain: (i) the spin-down band structure still maintains the semiconducting features, but its direct band-gap transforms into an indirect band-gap when compress strains are applied; in addition, the position of the conduction band minimum (CBM) drops, and the position of the valence band maximum (VBM) rises, which leads to a decrease in band-gap; (ii) the spin-up band still preserves the two band crossing points near the Fermi level as the strain is less than 9%; however, when the strain is larger than 9%, the band crossing points near the Γ-point disappear due to the shift in band, and the Weyl point along the Y → Γ path is shifted to the path of Γ → X. It can be seen that the biaxial strain has a significant modulating effect on the band structure of the MnNCl monolayer.

## 4. Conclusions

In summary, we have predicted a new 2D FM half-metallic material, i.e., the MnNCl monolayer, whose mechanical, magnetic, and electronic properties have been systematically studied based on first-principles calculations. Our calculations of the phonon spectrum and the elastic constant and molecular dynamics simulations (at 900 K) confirm that the structure of the MnNCl monolayer has superb stability, holding the possibility of synthesis in experiment. In mechanics, the MnNCl monolayer is a brittle material with large Young’s modulus and shear modulus and exhibiting strong anisotropy in the whole plane. For magnetic properties, the magnetic ground state of the MnNCl monolayer is the FM state with a large magnetic moment (6.16 μ_B_ per unit cell) and high Curie temperature (952 K). Moreover, the calculated MAE has a high anisotropy with a maximum value of 184.5 μeV, which is much higher than that of many reported two-dimensional materials. Interestingly, the mechanic moduli, magnetic moment, and MAE can all be enhanced or tuned by using strain. Our work identifies a novel high-temperature FM half-metal which should be promising for future spintronic application.

## Data Availability

The data presented in this study are available on request from the corresponding author.
